# Therapeutic Approach of Very Early-Onset Inflammatory Bowel Disease in a Loeys–Dietz Syndrome Child

**DOI:** 10.1097/PG9.0000000000000139

**Published:** 2021-12-10

**Authors:** Alina Opréa, Sophie Collardeau-Frachon, Sophie Heissat, Noel Peretti, Alain Lachaux, Rémi Duclaux-Loras

**Affiliations:** From the * Hospices Civils de Lyon, Hôpital Femme Mère Enfant, service de gastroentérologie, hépathologie et nutrition pédiatrique, Bron, France; † Hospices Civils de Lyon, Groupement Hospitalier-Est, service d’anatomopathologie, Bron, France; ‡ INSERM U1111, Centre International de Recherche en Infectiologie, Lyon, France.

**Keywords:** very early onset inflammatory bowel disease, Loeys–Dietz syndrome, ustekinumab

## Abstract

Heterozygous TGFBR2 loss-of-function mutation is an extremely rare cause of very-early onset inflammatory bowel disease (VEOIBD) as, so far, only three cases have been reported in the literature. VEOIBD therapeutic management remains a real challenge for clinicians. Here, we described an interesting new case of Loeys–Dietz syndrome presenting severe, very early intestinal inflammation associated with dysmorphic features, aortic arch tortuosity joint hyper laxity and severe scoliosis. TGFBR2 Sanger sequencing revealed a missense mutation c.1583G>A (p.Arg528His). As endoscopy confirmed a severe colitis, we chose a classical IBD therapeutic approach. We finally obtained remission under Ustekinumab (90 mg/6 weeks).

## INTRODUCTION

Transforming growth factor-β (TGF β) is a pleiotropic cytokine that modulates immune homeostasis, exhibiting both pro and anti-inflammatory properties as well as embryonic development (1). The main signaling pathway consists of phosphorylation and heterodimerization of type I and II TGF β receptors with subsequent phosphorylation and nuclear translocation of the receptor SMAD proteins (SMAD2 and SMAD3) (1).

Loeys–Dietz syndrome (LDS) is caused by heterozygous loss-of-function variants in TGF-βR1/-R2, TGF-β2:-β3, or SMAD2:SMAD3 (1–3). The syndrome is characterized by craniofacial anomalies (hypertelorism and cleft palate), skeletal involvement (joint hypermobility and scoliosis), cutaneous features (translucent skin), and arterial tortuosity/aneurysm (1). The clinical spectrum of gastrointestinal disorders in LDS includes food allergies, eosinophilic esophagitis, and inflammatory bowel disease (IBD) (1,4,5). Disruption of TGF-β signaling renders patients with LDS 10 times more susceptible to developing intestinal inflammation (6). Very early-onset inflammatory bowel disease (VEOIBD) is defined as IBD that occurs before 6 years of age. Early-onset disease may have a monogenic basis. To date, only 3 VEOIBD cases with TGFBR2 mutations have been reported in the literature (4,6). The rarity along with the lack of official VEOIBD treatment consensus brings numerous challenges to therapeutic management. We report the long-term management of a case of a VEOIBD patient with LDS undergoing a favorable clinical course under ustekinumab.

## CASE REPORT

We report the case of a male child born at 39 weeks gestation, 2480 g, with dysmorphic features including hypertelorism, cleft palate, translucent skin pectus excavatum, camptodactyly, median diaphragmatic hernia, and umbilical hernia. The association of aortic arch tortuosity, joint hyperlaxity, and precocious severe scoliosis lead to the suspicion of LDS. Genetic testing (Sanger sequencing method) identified a missense mutation in exon 7 of gene TGFBR2, c.1583G>A (p.Arg528His) at 1 year of age.

**FIGURE 1.**
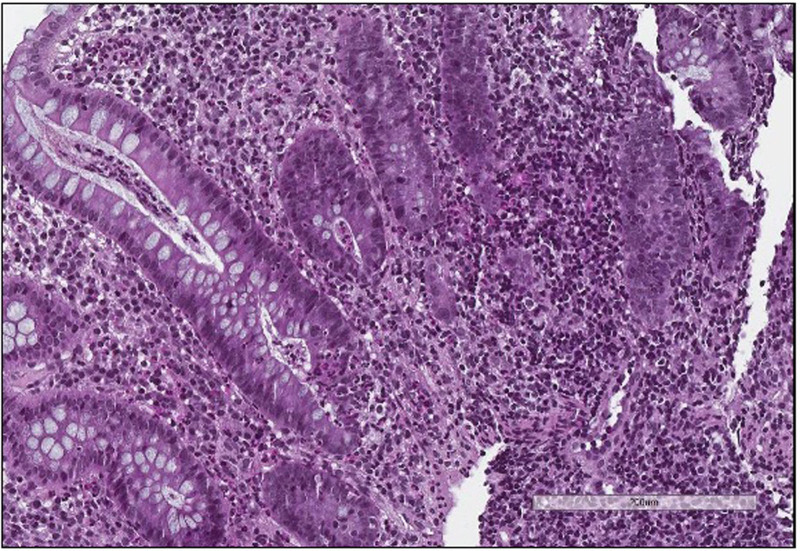
HES staining colonic biopsy of LDS patient. HES staining of patient’s colonic biopsy showing a severe chronic and active colitis, architectural disorder, crypt rarefaction, inflammatory infiltrate, crypt abscesses suggestive of IBD. IBD = inflammatory bowel disease; LDS = Loeys–Dietz syndrome.

At 5 months of age, the patient presented a failure to thrive and atopic dermatitis. At the age of 18 months, he presented idiopathic thrombocytopenic purpura. Concomitantly, he developed chronic bloody diarrhea with protein-losing enteropathy and intermittent fever. Initial laboratory studies showed elevated acute-phase reactants (erythrocyte sedimentation rate 50 mm/h and C-reactive protein 40 mg/L), microcytic anemia, severe hypoalbuminemia with hypergammaglobulinemia, and elevated total immunoglobulin E (672 kU/L). Diagnostic work-up excluded infection and revealed positive perinuclear antineutrophil cytoplasmic antibody (200 IU) along with elevated fecal calprotectin (5223 µg/g). Lymphocyte subpopulation counts were normal, except for elevated regulatory T cells (CD4+CD25+CD127−), 164/µL (44–60). Cytokine evaluation revealed elevated levels of tumor necrosis factor at 30 pg/mL (0–15). Plasmatic IL-6 and IL-10 were normal. At colonoscopy, all the colonic mucosa appeared diffusely ulcerated with multiple pseudopolyps. Biopsy specimens demonstrated severe pancolitis with active chronic inflammation and polymorph cellular infiltration with numerous eosinophils (Fig. 1). There was no evidence of granulomas. The colonoscopy was complicated by a colonic perforation and diaphragmatic hernia recurrence.

Initial treatment comprised of nutritional therapy along with oral azathioprine (2 mg/kg/d) and corticotherapy (2 mg/kg/d). As a result of relapses during prednisone tapering, anti-TNF-α biologic therapy (infliximab, 10 mg/kg) was started. Due to an anaphylactic shock during the second administration, the patient was switched to adalimumab (20 mg/2 weeks), on which he achieved transient response with a PCDAI dropping from 58 to 20 and decreased calprotectin from 3000 to 1200 µg/g. Despite posology incrementation of both azathioprine (3 mg/kg) and adalimumab (40 mg/2 weeks) at the age of 7 years, the disease was still active, exhibiting steroid dependence at 0.8 mg/kg/d. The PCDAI score was 32 and the fecal calprotectin was at 1809 µg/g. In addition, endoscopic showed moderate pancolitis. Colonoscopy was once again complicated by colonic perforation. Adalimumab was switched to Ustekinumab (90 mg/8 weeks). Two months later, even though the PCDAI score lowered to 17.5, due to the increase of fecal calprotectin (2204 µg/g) treatment was optimized by 6 weeks. After 18 months of treatment, the patient achieved a PCDAI score of 0, lowered fecal calprotectin at 54 µg/g, and reduced corticotherapy posology at 0.2 mg/kg/d with a ustekinumab serum trough level of 6 µg/mL with no side effects.

The parents are aware of this case report.

## DISCUSSION

We report the first case of VEOIBD caused by a TGFBR2 heterozygote mutation, p.R528H, to be successfully treated with ustekinumab.

TGF-β is a pleiotropic cytokine involved in embryonic development through the SMAD pathway. Patients present with multisystemic lesions characterized by vascular aneurysms, skeletal manifestations, craniofacial dysmorphia, and cutaneous defects. Patients with LDS are at high risk of aorta dissection and rupture, potentially leading to death and require close monitoring. Our patient presented with typical neonatal LDS features, including hypertelorism, cleft palate, translucent skin pectus excavatum, as well as aortic arch tortuosity.

Interestingly, due to the role of TGF- β in immunoregulation, LDS patients are predisposed to allergic and inflammatory disease such as asthma, eczema, and reactions to food allergens. Moreover, IBD incidence is higher in LDS patients compared to the general population (6). Indeed, IBD in LDS is estimated at around 4.3%. However, only 3 patients with LDS and VEOIBD presenting with intestinal failure before 6 years have been reported. Similar to our patient, digestive disease began before 2 years of age (Table 1). Two patients presented with isolated colitis and one patient presented with colitis associated with duodenum ulcerations (Table 1). Histologic analysis was not specific, showing eosinophilic infiltration in the upper and lower part of the GI tract correlated to an allergic process (Table 1).

**TABLE 1.** Reported patients suffering from Loeys–Dietz syndrome combined with very-early-onset inflammatory bowel disease

Age onset	Sex	IBD type	Clinical presentation	Associated GI disorder	dysmorphic features	Cardiovascular disorders	Histology	Steroids	losartan	Immuno modulators	Outcome
3 mo (6)	F	UC	Hematochezia	CMPA	Craniosynostosis Hypertelorism Bifid uvula Submucous cleft Arched palate		Lamina propria eosinophilia Microscopic pan colitis Basal plasmocytosis, Paneth cell metaplasia		−	6 – MP (TR) Sirolimus (R)	Dead
13 mo (4)	F	UC	Failure to thrive Bloody diarrhea Fever	two perianal extrasphincteric abscesses	Hyperthelorism Proptosis Blue sclerae Joint hyperlaxity Arachnodactyly Cervical spine instability	Mild dilation of the ascending aorta	Lamina propria eosinophilia Lymphoplasmocellular infiltrate Crypt abcesses Paneth cell metaplasia	Cortico-dependency	+	Azathioprine + ciclosporine (TR)	Alive
26 mo (4)	M	CD	Failure to thrive Bloody diarrhea Fever		Hyperthelorism Blue sclerae Bifid uvula Arachnodactyly Joint hyperlaxity Pectus carinatum Pes valgus	Dilation of the aortic root	Lymphoplasmocellular Infiltrate Crypt abcesses	Cortico-dependency	+	Azathioprine (TR)	Alive
18 mo	M	CD	Failure to thrive Bloody diarrhea Enteropathy	CMPA	Hypertelorism, Cleft palate, Translucent skin Pectus excavatum, Camptodactyly, Umbilical hernia	Dilation of the aortic root	Severe pan colitis with active chronic inflammation Eosinophilia	Cortico- dependency	−	Azathioprine (TR)	Alive

6-MP = 6-mercaptopurine; CD = Crohn disease; CMPA = cow milk protein allergy; GI = gastrointestinal; NR = nonresponsive; R = remission; TR = transient response; UC = ulcerative colitis.

Management of VEOIBD is challenging for the clinician as patients respond poorly to standard immunosuppression (7). In our case, we treated the patient with nutritional first-line therapy followed by corticosteroids and finally biologics. Indeed, ustekinumab was reported as efficient in anti-TNFα resistant adult and pediatric CD patients but never reported in VEOIBD (7). In our patient, ustekinumab allowed a dramatic decrease in PCDAI and fecal calprotectin within 5 months. The impact of TGFBR2 mutations on the immune response is complex. Patients presented with increased TH2 immune response associated with increased pSMAD3. So far, the TH17 response in TGFBR2 patients has never been evaluated. We can speculate that intestinal inflammation could be due overactivation of TH17 cells in our patient, resulting in high level of IL-23 secretion. As an anti-p40, subunit of IL-23, ustekinumab could reduce the level of intestinal inflammation. Sirolimus, an immunosuppressant that inhibits mTOR, has also been reported as efficient in dysimmune enteropathies (8) and was successfully used in one patient with a TGFBR2 mutation. Improvements within 2 weeks of treatment were observed along with normalization of inflammatory markers (6). We recommend that VEOIBD in TGFBR2 should be treated as a step-by-step therapeutic approach with azathioprine associated with steroids followed by biologics, including anti-TNF-α and ustekinumab. Sirolimus should be introduced in the event of biologic failure.

Our case confirmed that LDS is associated with VEO-IBD resistance to standard immunosuppression and highlights the role of TGF-β signaling in gut homeostasis.
